# *Morinda citrifolia* (Noni) Juice Augments Mammary Gland Differentiation and Reduces Mammary Tumor Growth in Mice Expressing the Unactivated c-*erb*B2 Transgene

**DOI:** 10.1155/2012/487423

**Published:** 2012-04-26

**Authors:** William P. Clafshenkel, Tracy L. King, Mary P. Kotlarczyk, J. Mark Cline, Warren G. Foster, Vicki L. Davis, Paula A. Witt-Enderby

**Affiliations:** ^1^Graduate School of Pharmaceutical Sciences, Duquesne University, Pittsburgh, PA 15282, USA; ^2^Division of Clinical, Social, and Administrative Sciences, Mylan School of Pharmacy, Duquesne University, Pittsburgh, PA 15282, USA; ^3^Department of Pathology, Wake Forest University School of Medicine, Winston-Salem, NC 27127, USA; ^4^Department of Obstetrics & Gynecology, McMaster University, Hamiton, ON, Canada L8S 4K1; ^5^Barnes Center, Center for Applied Research & Intellectual Property Development, Clarion University, 840 Wood Street, Clarion, PA 16214-1232, USA

## Abstract

*Morinda citrifolia* (noni) is reported to have many beneficial properties, including on immune, inflammatory, quality of life, and cancer endpoints, but little is known about its ability to prevent or treat breast cancer. To test its anticancer potential, the effects of Tahitian Noni Juice (TNJ) on mammary carcinogenesis were examined in MMTV-*neu* transgenic mice. Mammary tumor latency, incidence, multiplicity, and metastatic incidence were unaffected by TNJ treatment, which suggests that it would not increase or decrease breast cancer risk in women taking TNJ for its other benefits. However, noni may be useful to enhance treatment responses in women with existing HER2/*neu* breast cancer since TNJ resulted in significant reductions in tumor weight and volume and in longer tumor doubling times in mice. Remarkably, its ability to inhibit the growth of this aggressive form of cancer occurred with the mouse equivalent of a recommended dose for humans (<3 oz/day). A 30-day treatment with TNJ also induced significant changes in mammary secondary ductule branching and lobuloalveolar development, serum progesterone levels, and estrous cycling. Additional studies investigating TNJ-induced tumor growth suppression and modified reproductive responses are needed to characterize its potential as a CAM therapy for women with and without HER2^+^ breast cancer.

## 1. Introduction

Approximately 41% of women are utilizing complementary and alternative medicine (CAM) forms of medicine to manage their breast cancer [1, 2], including products from the *Morinda citrifolia *(noni) plant. An edible and medicinal tropical plant, *Morinda citrifolia, *has been used for over 2000 years by Polynesian cultures as an herbal remedy for infection, arthritis, diabetes, asthma, hypertension, and pain [3]. All parts of the plant including the roots, bark, stems, flowers, leaves, and fruit are components in various combinations of 40 known and recorded herbal remedies [3], with the fruit being the most researched [4]. The popularity of noni has spread from Polynesian and Hawaiian cultures with its global introduction in the early 1990s [5], and its products are now readily available in health food stores and on the Internet.

Several studies reported that noni has multiple cancer protective properties. Oncostatic activities related to cancer prevention include reductions in TPA- or EGF-induced cell transformation [6], reduction in DMBA-induced DNA damage and lesion formation [7, 8], and concentration-dependent free-radical scavenging effects [8–10]. Similarly, additional anticancer activities, including antiangiogenic [11] and cancer cell-selective cytotoxic properties [12, 13], suggest its potential as a treatment to inhibit tumor growth and progression. Although only a few studies have tested the cancer inhibitory actions of noni *in vivo*, one recent study demonstrated that noni fruit powder had both preventative and treatment efficacy on rat esophageal cancer induced by N-nitrosomethylbenzylamine [14]. For breast cancer, although its oncostatic properties suggest that noni would inhibit carcinogenesis, there have been no preclinical studies testing its potential to influence mammary tumor development, except for an abstract reporting that noni inhibits the initiation stage of DMBA-induced mammary cancer in rats [7]. Moreover, women are utilizing noni for the prevention and treatment of breast cancer and as a secondary course of treatment following conventional chemotherapy [15], even though its ability to influence breast cancer development or progression has not been determined. Therefore, in the present study, the impact of noni fruit juice administration on mammary tumor development and tumor growth was investigated in MMTV-*neu* transgenic mice. This model expresses the unactivated rat *neu* (c-*erb*B2) gene under the transcriptional control of the mouse mammary tumor virus (MMTV) promoter. This mouse model mimics many features of HER2/*neu* ^+^ breast cancer, including stochastic tumor onset, focal tumors arising near hyperplastic tissue, a long latency period, estrogen-dependent tumor development, and metastatic progression to the lungs [16–18]. As no studies have investigated the influence of noni on metastatic progression in any type of cancer, this study with MMTV-*neu* mice will examine its ability to influence the spread of cancer outside the mammary gland.

Seven case reports of liver damage have been reported between 2005 and 2011 associated with noni use alone or in combination with other CAM or traditional medications [19–22]. Since noni is a common herbal therapy with sales in U.S.A estimated over $250 million in 2005 [4], these few reports suggest that hepatotoxicity is a rare event. In addition, preclinical studies suggest that noni therapies may have hepatoprotective properties [23, 24]. Concerns with kidney function have also arisen due to a case report documenting elevated potassium levels in a patient with chronic renal insufficiency taking noni juice [25]. Therefore, to determine if long-term administration of a recommended dose (equivalent to less than 3 ounces/day in humans) shows any liver or kidney toxicity, serum markers and histopathology were examined in aged mice chronically treated with noni juice.

In this study, the influence of a noni fruit juice, Tahitian Noni juice (TNJ), on mammary tumor development, growth, and metastatic progression in the MMTV-*neu* mice was investigated. Additionally, to identify potential responses that could influence mammary tumorigenesis, differentiation in the normal mammary gland, serum hormone levels, and estrous cycling was examined in sexually mature female mice treated for 1 month, prior to tumor onset.

## 2. Materials and Methods

### 2.1. Noni Juice Preparation and Dosage

The Tahitian Noni Juice or TNJ brand of noni juice (Tahitian Noni International, Utah) was utilized in this study as it supplies the noni fruit as a puree with no chemical fillers, thickeners, or preservatives. The puree from ripe noni fruit harvested in Tahiti used in TNJ contains many nutrients and phytochemicals [26], including iridoids. TNJ is standardized to contain 30 mg to total iridoids/60 mL serving. Moreover, TNJ is one form of noni which has been frequently studied [3, 8, 23, 27–30] and which is available for use in women. Administration of TNJ in the drinking water was used to mimic oral consumption by women. The concentration used in this study, 10% TNJ v/v added in the drinking water, would result in the daily consumption of 0.35 mL of TNJ, based on a mouse drinking on average 3.5 mL of water daily. The equivalent human dose would be between 1.5 to 2.75 oz (45–80 mL) based on daily caloric intake (1800 kcal/day for women versus 14.4 kcal/day for mice) to account for metabolic differences or average body weight for each species, respectively. Different lots of TNJ were used across the course of the study. The juice was refrigerated after opening. TNJ (10% v/v in UV-purified drinking water) or vehicle control (UV-purified drinking water) were provided *ad libitum *to animals to mimic women taking TNJ or not. Water bottles were changed twice weekly.

### 2.2. Tumor Animal Study

All animal work was approved by the Duquesne University Institutional Animal Care and Use Committee (IACUC). The hemizygous female MMTV-*neu* mice harboring the *neu *protooncogene were bred from dizygous transgenic males (FVB/N-Tg(MMTVneu)202Mul/J [17]) and wild-type FVB/N females (Jackson Laboratory, Bar Harbor, ME). The female progeny was randomized into control and 10% TNJ groups at weaning and kept on a standard 12 hour light/12 hour dark lighting schedule. Both groups received an isoflavone-free diet from conception to death to eliminate potential confounding effects from these phytoestrogens. The diet is a modification of AIN-93G using corn oil (Harlan Teklad, Madison, WI, USA) [31]. Treatment was started after sexual maturity (2 months of age) to correlate with noni use in adult women and continued until death. Tumor-free mice were necropsied at the maximum age of 14 months, unless due to maximum tumor burden, ulcerated tumors, restricted movement, or illness.

### 2.3. Tumor Development, Kinetics, and Progression

Mammary tumor onset was determined by weekly palpations of mammary glands starting at 4 months of age. In growing tumors, caliper measurements on 2 dimensions were used to calculate mammary tumor volume using a modified ellipsoid formula: mm^3^ = 1/8[4/3*π* (large diameter)(small diameter)^2^]. During necropsy, resected mammary tumors were measured in 3 dimensions to calculate tumor volume using the ellipsoid formula: mm^3^ = 1/8 [4/3*π* (diameter *x*)(diameter *y*)(diameter *z*)]. Mammary tumor doubling time (DT) was calculated as: DT = (*T* − *T*_0_)·[log⁡⁡2/(log⁡⁡*V* − log⁡⁡*V*_0_)] [32], where *T* − *T*_0_ represents the length of time (days) between two measurements, *V*_0_ is the tumor volume at the starting point (*T*_0_), and *V* is the tumor volume at the end time point (*T*) of the time period.

### 2.4. Tissue Histopathology

Histopathology was completed by a board-certified veterinary pathologist (J.M.C.) blinded to the treatment groups on hematoxylin and eosin-stained sections (Mass Histology Services, Worcester, MA, USA). Renal and hepatotoxicity and vascularity and necrosis in mammary tumor sections were qualitatively assessed. Micrometastases were quantified as the total number of emboli or invasive lesions detected in a lung section as previously described [31].

### 2.5. Identification and Quantification of Blood Vessels in Tumor Sections

Solid mammary tumors fixed with cold 4% paraformaldehyde were immunostained for von Willebrand factor. Tumor sections were incubated with the primary antibody (rabbit anti-von Willebrand factor; 1 : 100; Millipore, Ontario, Canada), with secondary antibody (biotinylated anti-rabbit IgG; 1 : 500; Vectastain Elite ABC kit, Vector Laboratories, Ontario, Canada), and with diaminobenzidine, followed by counterstaining with Harris hematoxylin. From each section, ten regions of interest were randomly identified using a grid overlay and immunopositive cells counted by an investigator blinded to the treatment groups.

### 2.6. Pretumor Animal Study

Additional hemizygous MMTV-*neu *female mice were randomized into the control and 10% TNJ treatment groups (10/group) and treated as above for 1 month. Estrous cycling was monitored by daily morning vaginal lavage for the 30 days of treatment. Cells were stained with Dif-Quick Stain Kit (IMEB Inc., San Marcos, CA) and cycle stage was determined by established criteria [33]. Mice were necropsied on the first day in estrus after the 30-day treatment, and cycle stage was reconfirmed immediately prior to euthanasia.

### 2.7. Steroid Hormone Levels and Mammary Gland Whole Mounts

Serum 17*β*-estradiol and progesterone were assessed using the 17*β*-estradiol double antibody radioimmunoassay kit (Siemens, Los Angeles, CA, USA) and the progesterone Coat-A-Count radioimmunoassay kit (Siemens, Los Angeles, CA, USA) on animals in estrus with sufficient serum for both assays. Whole mounts were prepared on inguinal mammary glands fixed in cold 4% paraformaldehyde by carmine alum staining. Fixed glands were covered in carmine alum stain (4 mM carmanic acid/0.1 mM aluminum potassium sulfate) overnight at 4°C, rinsed in a graded series of ethanol washes, defatted and cleared with toluene, and stored in methylsalicylate. Mammary gland morphology was quantified from photomicrographs using NIH Image J software and based on guidelines previously reported [34]. The percentage of ductal tree occupation in the mammary fat pad was calculated as the ratio of the distance from the center of the inguinal lymph node to the terminal end bud (TEB) by the distance from the center of the inguinal lymph node to the edge of the mammary fat pad. The ratio was calculated to control for difference in mammary gland shape and size between animals. The number of TEBs was calculated as those terminal structures that were greater than or equal to 0.015 mm^2^. The number of secondary ducts was quantified by locating larger, distinct primary ducts and counting the number of secondary branches along a 1 mm distance. Ductal thickness was reported as the average of the transverse distance measured across three separate primary ducts. The average number of lobuloalveolar structures was determined by quantifying lobuloalveolar structures in four separate 16 mm^2^ areas.

### 2.8. Statistical Analysis

Categorical variables were statistically analyzed using the Fisher's exact test. The statistical difference between group means was analyzed with the Student's *t*-test when the distribution of the data was approximately symmetric and with the Mann-Whitney test when the distribution of the data was skewed. Survival curves were compared by the Gehan-Breslow-Wilcoxon test. Analyses were performed using GraphPad Prism 5.0 software with <0.05 *P*-value considered significant.

## 3. Results

### 3.1. TNJ Influence on Mammary Tumor Development and Growth

**Figure 1:**
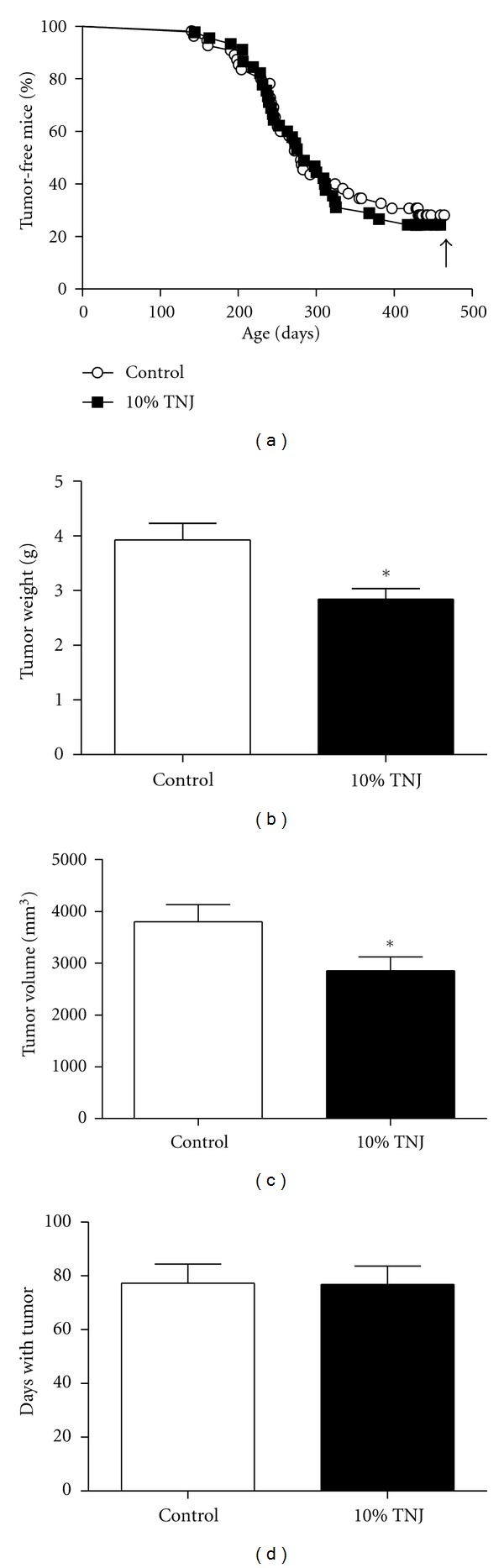
Chronic administration of 10% TNJ affects mammary tumor size, but not tumor development, in female MMTV-*neu* mice. (a) Survival curves show mammary tumor incidence with age. The percent of tumor-free MMTV-*neu *females for the control (*n* = 55) and TNJ-treated (*n* = 45) groups up to the maximum age of 14 months are not significantly different (Gehan-Breslow-Wilcoxon test). Black arrow indicates maximal primary mammary tumor incidence at approximately 14 months of age. Control: 72.2%; 10% TNJ: 75.6%. (b) The average weight of the first detected mammary tumor for TNJ-treated mice (*n* = 30) was found to be significantly reduced (*P* < 0.010, Mann Whitney test) compared to control mice (*n* = 30). Only solid tumors that remained separate from other tumors and had time to grow to a maximum volume of at least 800 mm^3^ were used for the volume and weight calculations. (c) The average, noncystic mammary tumor volume for the first detected tumor in TNJ-treated mice (*n* = 30) was smaller than for the control group (*n* = 30), *P* < 0.038, Mann Whitney test. Tumor volume was measured on 3 dimensions after dissection. (d) To correlate with length of time each tumor had to grow for the first detected mammary tumor, the time between detection and death was determined for the mice in (b) and (c). The same animals were used to evaluate both tumor volume and weight (*n* = 30 for control and *n* = 30 for TNJ). No significant difference was detected by the Mann Whitney test. Mean ± SEM are shown; TNJ: Tahitian Noni Juice; *indicates significance, *P* < 0.05, by the Mann-Whitney test.

Mammary cancer development, growth, and progression to metastatic disease in the MMTV-*neu *mouse model were assessed in control and 10% TNJ-treated animals until a maximum age of 14 months. The mean age of mammary tumor latency (259.8 ± 10.6 days, *n* = 39, for control versus 266.1 ± 9.9 days, *n* = 35, for TNJ-treated animals) and overall mammary tumor incidence (72.2% control, 75.6% TNJ-treated animals) were unaffected by TNJ treatment. Survival curves for the percent tumor-free animals with age did not result in a significant difference between the groups (Figure 1(a)). Mammary tumor multiplicity was also similar between groups with 2.51 ± 0.25 versus 2.89 ± 0.24 mammary tumors per tumor-bearing mouse for the control (*n* = 43) versus the TNJ-treated group (*n* = 44), respectively. Thus, no effect of 10% TNJ treatment was observed on mammary tumor development in the MMTV-*neu* mouse model.

To investigate the impact of TNJ on tumor growth, the mammary tumor with the earliest onset in each mouse was analyzed. Solid mammary tumors were examined for size and growth analyses as there is intrinsic variability in tumor volume measurements of cystic tumors, tumor weight and volume can be affected by loss of tumor fluid during excision, few tumors were cystic in this study, and solid tumors best represent the efficacy of TNJ against dense, heterogeneous populations of tumor cells. The average mammary tumor weight (Figure 1(b)) and volume (Figure 1(c)) were both significantly lower in the 10% TNJ-treated group. Moreover, the time between tumor detection and death was not significantly different between the treated and control groups (Figure 1(d)). These data indicate that the decreased tumor volume and weight for the TNJ-treated mice was related to the treatment and not to the tumors in the control group having more time to grow.

**Figure 2:**
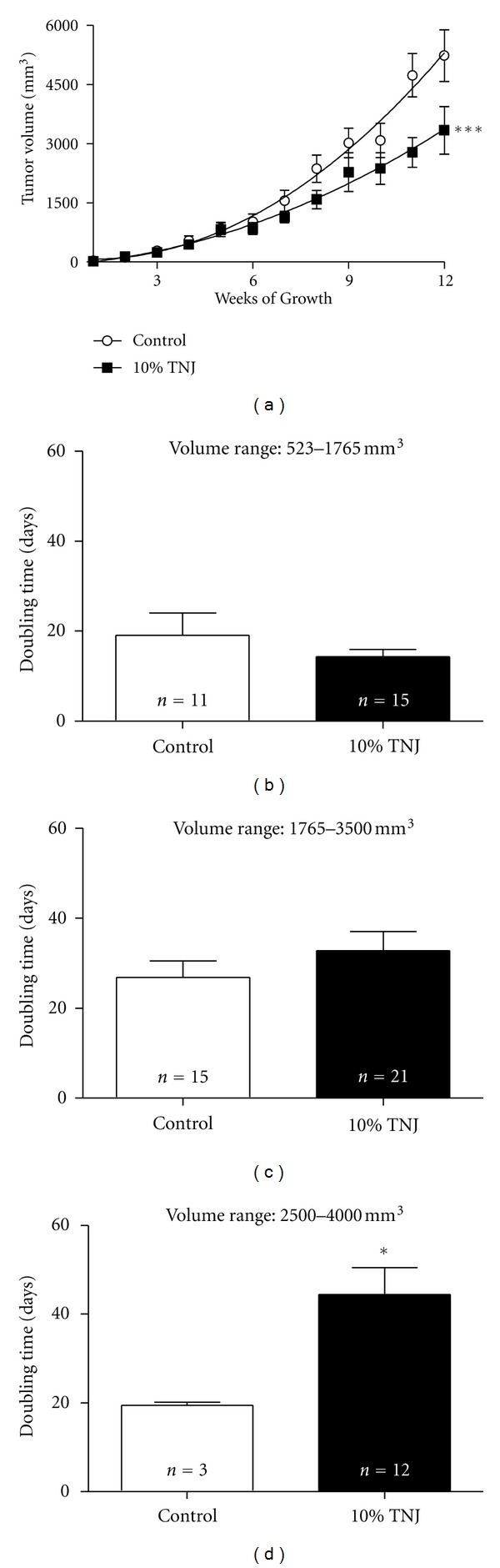
Slower tumor growth kinetics in live animals was observed in the 10% TNJ-treated MMTV-*neu* animals than in the control group for large tumors. (a) Growth of spontaneously developed mammary tumors was measured weekly with calipers on 2 dimensions. The first solid tumor that was detected ≤5 mm in diameter in animals for the control (*n* = 9) and 10% TNJ (*n* = 13) groups was compared. These curves were significantly different, *P* < 0.0001, as determined by nonlinear regression analysis in Graphpad Prism 5. Due to the inherent heterogeneity of tumor behavior in this model related to the unique mutations that induce the mammary cancer [35], very rapidly and slowly growing tumors were excluded. The criteria for exclusion included tumors that grew for less than 2 months (9 weeks) since detection or did not reach a maximum volume of 900 mm^3^ by 12 weeks after detection. (b) Doubling time for solid mammary tumors with initial volume ≥532 mm^3^ and that grew to the maximum volume of ≥1765 mm^3^ with at least 2 weeks between these 2 measurements was used to assess the growth of tumors in the small range. The number of mice examined is shown in the bars for each group. (c) For intermediate size tumors, the doubling time for tumors growing for 2 weeks or longer within the volume range of 1765–3500 mm^3^ is shown with the number of mice per groups shown in the bars. (d) 10% TNJ increased mammary tumor doubling time for the large volume range (*P* = 0.036; 2500–4000 mm^3^). Only 3 tumors in the control group met the criteria of 2 weeks between the volumes for this range due to the rapid growth of their larger tumors. For this figure, only mice with noncystic mammary tumors that remained separate to allow accurate measurement were included. Mean ± SEM; TNJ: Tahitian Noni Juice; *indicates significance, *P* < 0.05 as determined by Mann-Whitney test.

Although the final tumor weight and volume indicate growth suppression, these measurements do not indicate how the size changed with time in the live animal; therefore, weekly growth patterns and doubling time were compared between the control and TNJ groups. Weekly growth profiles for control and TNJ-treated mammary tumors show significant size reduction with noni treatment (Figure 2(a)). Separation of the curves occurs around 9 weeks after tumor detection when the average volume of the control tumors is approximately 2500 mm^3^, suggesting that inhibition of tumor growth by TNJ compared to the control group occurs as the tumor volume increases. Therefore, tumor doubling time was determined on specific ranges of tumor volumes between 523 and 4000 mm^3^ (approximately 10 × 10 mm to 20 × 20 mm); these initial and final sizes correlate to clinically detectable tumor sizes of 1 cm and 2 cm which are associated with stage 1 breast cancer. Doubling time for several volume ranges was analyzed for small (523–1765 mm^3^, 10 × 10 mm to 15 × 15 mm), intermediate (1765–3500 mm^3^, 15 × 15 mm to 19 × 19 mm), and large tumor sizes (2500–4000 mm^3^, 17 × 17 mm to 20 × 20 mm) in mice (Figures 2(b)–2(d). TNJ treatment had significant growth inhibitory effects within the large volume range of 2500–4000 mm^3^ (Figure 2(d)), which corresponds to the size of tumors when the growth curves for the control and TNJ diverge (Figure 2(a)). Tumor doubling times for the entire size range approached significance for reduced tumor growth with TNJ treatment (523–4000 mm^3^, *n* = 9 and 17, *P* = 0.067; data not shown). These results suggest that TNJ treatment results in significant growth inhibitory effects, which likely account for the significant reductions in mammary tumor weight and volume at necropsy noted with chronic TNJ treatment (Figures 1(b) and 1(c)).

**Figure 3:**
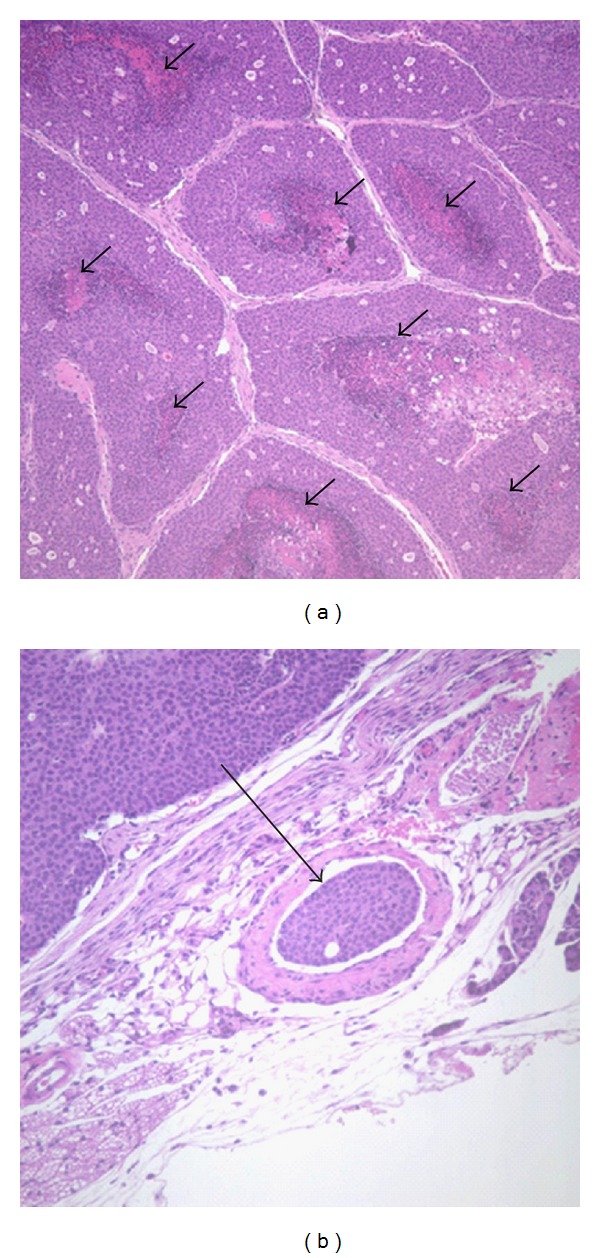
Central necrosis and tumor intravasion are evident in histological MMTV-*neu* mammary tumor sections. (a) A mammary tumor from the TNJ-treated group is shown with comedo pattern of central necrosis within each region, magnification at 100x. Black short arrows designate necrosis in multiple regions of the tumor. (b) Control group mammary tumor with vascular invasion of tumor cells to form an embolus within a blood vessel (designated by the black long arrow) is shown, magnification at 200x.

**Figure 4:**
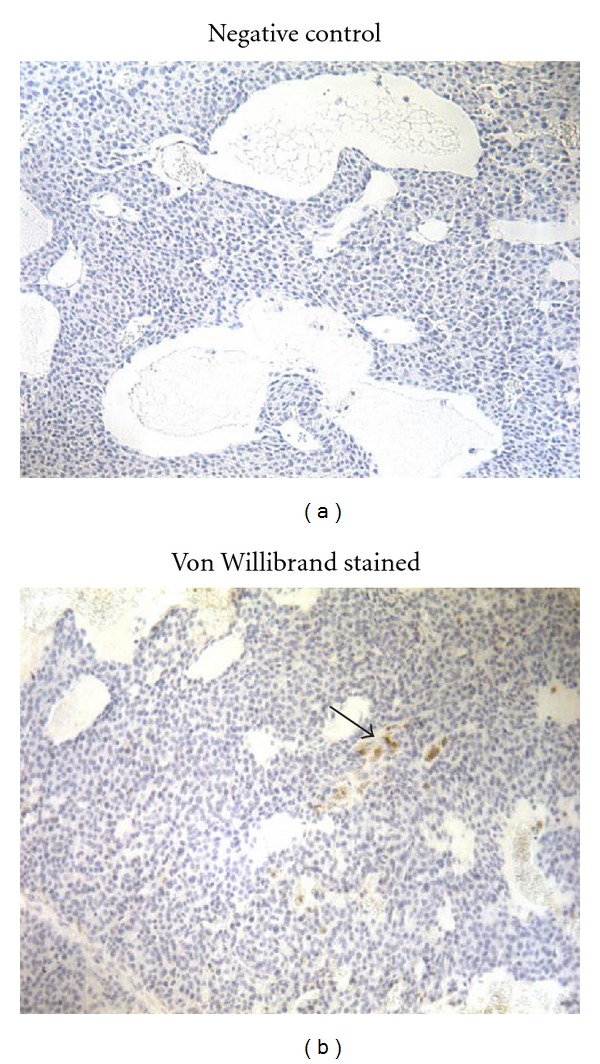
Example sections for the negative control (no primary antibody) and for immunostaining with anti-von Willebrand factor in MMTV-*neu* mammary tumors. (a) Staining was absent in a negative control section. The primary antibody was replaced with nonimmune serum (1 : 100) for negative control slides. (b) Immunohistochemistry resulted in isolated clusters of epithelial cells (arrow) in sections of mouse mammary tumors stained with anti-von Willebrand factor antibody (1 : 100). Sections were counterstained with Harris hematoxylin. Original magnification 200x.

The prevalence of necrosis in mammary tumor sections (Figure 3(a)) was determined by a board-certified veterinary pathologist (J.M.C.), with a nearly significant increase in central necrosis detected with TNJ treatment (*P* < 0.064, Fisher's exact test; Table 1). To determine if a potential cause of the necrosis could be due to poor vascularization of the tumors, blood vessels were quantified in tumor sections by immunostaining for von Willebrand factor (Figure 4). The average number of immunopositive blood vessels in the tumors did not vary significantly between groups (Table 1), although this may be attributed to a small sample size.

Histopathology identified tumor cells invading into the blood vessels within the tumor (Figure 3(b)); fewer tumors in TNJ-treated mice showed evidence of vascular invasion compared to the control group, which approached significance (*P* < 0.072, Fisher's exact test; Table 1). As vascular invasion is a crucial step in tumor cells spreading outside the mammary gland, these data suggest that TNJ may reduce metastatic progression. However, metastatic incidence for tumor-bearing mice and the average number of lesions in mice with micrometastases (with and without extravasion) were not statistically different for the control and 10% TNJ groups (Table 2). These results suggest that while 10% TNJ treatment impeded growth of the mammary tumors, it did not prevent metastatic progression to the lungs in the MMTV-*neu* mice, despite less vascular invasion within the mammary tumors (Table 1).

**Figure 5:**
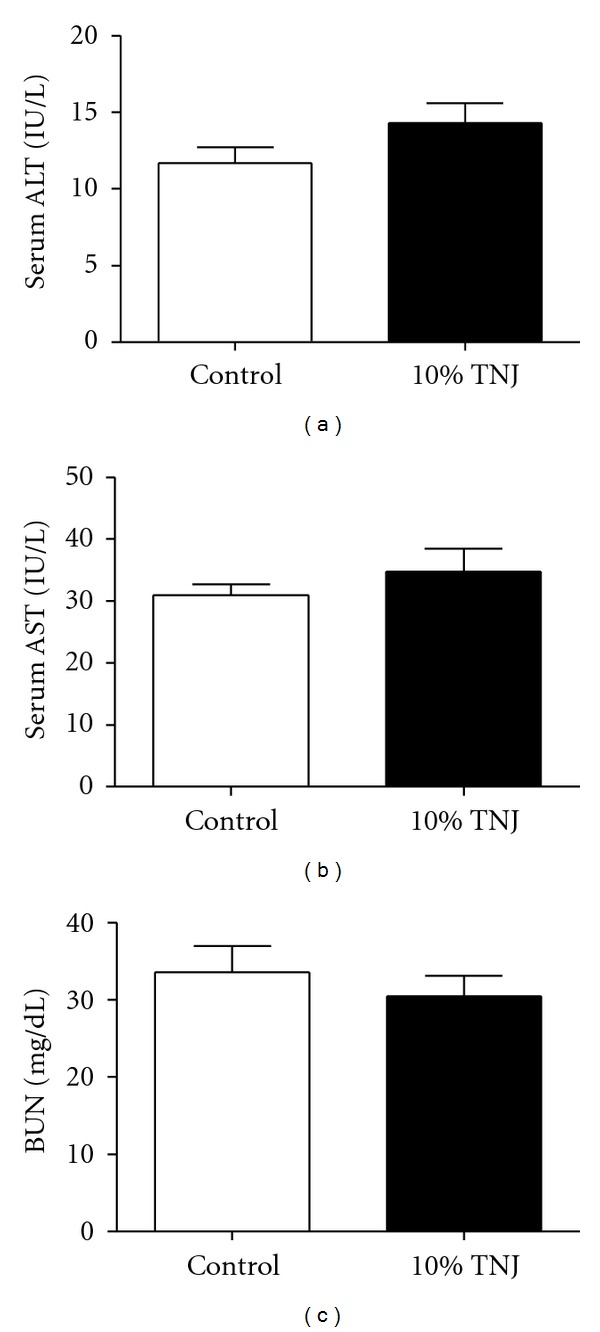
Chronic administration of 10% TNJ does not elevate serum levels of markers for hepatic renal toxicity in 14-month-old female MMTV-*neu* mice. (a) Serum alanine aminotransferase (ALT), a hepatic serum marker, in control, *n* = 7, and 10% TNJ-treated, *n* = 7, mice. (b) Serum aspartate aminotransferase (AST), a marker of liver damage, in control, *n* = 6, and 10% TNJ, *n* = 6, groups. (c) Serum blood nitrogen urea (BUN), a renal function marker, in control, *n* = 6, and 10% TNJ, *n* = 2, groups. Only animals necropsied within 48 hours of the assay were used to analyze BUN levels. No significance was detected for any of the markers, *P* > 0.05; *P *values determined by Student's *t*-test. Mean ± SEM are shown; TNJ: Tahitian Noni Juice.

The possibility of TNJ-induced toxicity was assessed in aged mice consuming the equivalent of a recommended dose in women for most of their adult life. Serum levels of the hepatic enzymes, aspartate transferase (AST) and alanine transferase (ALT), were examined in the TNJ-treated mice, since these are considered markers of liver damage in humans and both were elevated in the case reports on hepatotoxicity with noni use [19–22, 36, 37]. In addition, serum blood urea nitrogen (BUN), a marker of renal function, was assessed. None of these three markers were abnormally elevated or disproportionate to levels in the control group (Figure 5). These results were supported by the absence of toxin-induced damage to tissue ultrastructure in sections of liver and kidney from the 10% TNJ-treated group (data not shown). Thus, long-term administration of TNJ in the MMTV-*neu* model did not have any detectable adverse effects on the liver and kidney tissues or markers assessed in this study.

### 3.2. Reproductive Endpoints in Young TNJ-Treated Mice

**Figure 6:**
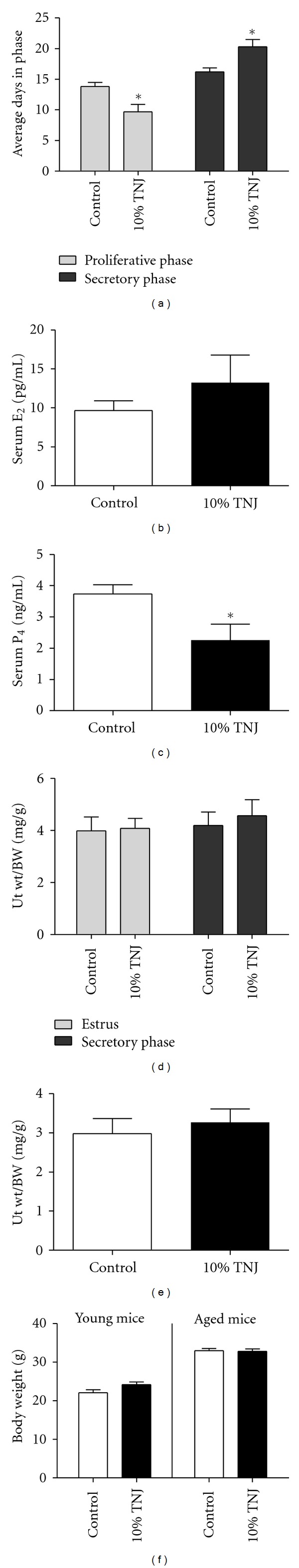
TNJ treatment results in more days in the secretory phase of the estrous cycle over a 30-day period and in reduced serum progesterone levels. (a) Effects on estrous cycling were analyzed in virgin, tumor-free MMTV-*neu* mice over thirty days of treatment with 10% TNJ. The time spent in the proliferative (follicular) phase decreased and increased in the secretory (luteal) phase in females treated with 10% TNJ compared to the control animal over the 30-day period (between ages 2 and 3 months), *P* = 0.002 by the Mann-Whitney test (*n* = 10 for each group). Vaginal smears in the morning were used to determine secretory phase stages (diestrus and metestrus) and proliferative phase stages (proestrus and estrus). (b) Serum 17*β*-estradiol (E_2_) levels for control (*n* = 5) and 10% TNJ-treated females (*n* = 6) were measured by radioimmunoassay in virgin, tumor-free 3-month-old MMTV-*neu *mice that were in estrus at necropsy. Serum levels were not significantly different between groups. (c) Serum progesterone (P_4_) levels for control (*n* = 7) and 10% TNJ-treated females (*n* = 8) were measured in the same mice analyzed for 17*β*-estradiol levels (in estrus). Serum progesterone levels, as analyzed by Student's* t*-test, were significantly reduced in mice receiving 10% TNJ for 1 month (*P* = 0.029). (d) For tumor study mice in estrus or in the secretory phase (metestrus and diestrus) at the time of necropsy, uterine wet weight normalized to body weight (Ut wt/BW) was not significantly different for the control and 10% TNJ-treated groups. Control estrus (*n* = 12) and secretory phase (*n* = 31); TNJ estrus (*n* = 15) and secretory phase (*n* = 20). Mice used for uterine weights in the tumor study were, on average, around 1 year of age and were euthanized for maximum age or tumor size. (e) For the 3-month-old females in the pretumor study, uterine wet weight normalized to body weight (Ut wt/BW) for mice in estrus just prior to necropsy were not significantly different for either treatment group; control (*n* = 6), TNJ (*n* = 9). (f) Body weights after euthanasia for young animals treated for 1 month (3-month-old mice, *n* = 10) or aged mice (*n* = 58, control; *n* = 55, TNJ) in the tumor study in TNJ-treated and control groups were significant by 2-way ANOVA for age (*P* < 0.001), but not treatment. Mean ± SEM are shown; TNJ: Tahitian Noni Juice; *indicates significance, *P* < 0.05 by Mann Whitney or Student's *t*-test.

The effects of *Morinda citrifolia *(noni) juice on estrous cycling was assessed in MMTV-*neu* mice treated for thirty days, starting approximately 5 weeks after the onset of puberty. While treatment with 10% TNJ did not result in significant changes in the time spent in estrus (9.3 ± 0.6 days, *n* = 10 for control and 7.9 ± 3.1 days, *n* = 10 for TNJ), these mice on average spent approximately one-third of the 30 days in the proliferative phase and two-thirds in the secretory phase of the cycle (Figure 6(a)). In contrast, the control group spent approximately the same number of days in each phase. Alteration in the cycling pattern over the thirty-day period suggests that TNJ treatment may impact the hormonal pathways which regulate the estrous cycle.

Serum levels of the ovarian hormones, 17*β*-estradiol and progesterone, were analyzed in the 3-month-old mice in estrus. Serum levels of 17*β*-estradiol were not significantly affected by the 30-day treatment, but there was a trend towards elevated levels with 10% TNJ (Figure 6(b)). Circulating levels of progesterone from the same animals were significantly reduced in the 10% TNJ-treated group compared to control levels (Figure 6(c)). As the decreased progesterone levels occurred during the phase of the cycle when its levels are low (estrus), these results suggest that the baseline levels of progesterone are modified by TNJ administration.

Due to changes in the estrous cycling and serum progesterone levels, uterine stimulation was investigated in the mice treated short term (30 days) or long term (up to 12 months) with TNJ. The lower progesterone levels for mice in estrus (Figure 6(c)) could result in higher uterine wet weights in this phase of the cycle due to less of this uterine protective hormone available to counteract the estrogen-induced stimulation, especially with the trend to higher 17*β*-estradiol levels (Figure 6(b)). However, no changes in uterine weight were observed in the aged (Figure 6(d)) or young mice (Figure 6(e)) in estrus. In addition, at times of low estrogen (secretory phase), no increases in uterine weight were detected after long-term TNJ treatment (Figure 6(d)). Therefore, the significant changes in progesterone levels and cycling did not result in an observable effect on this highly hormone-responsive tissue. Body weights in the young and aged mice were also not significantly different in the 1-month and long-term TNJ-treated mice compared to the control group (Figure 6(f)).

**Figure 7:**
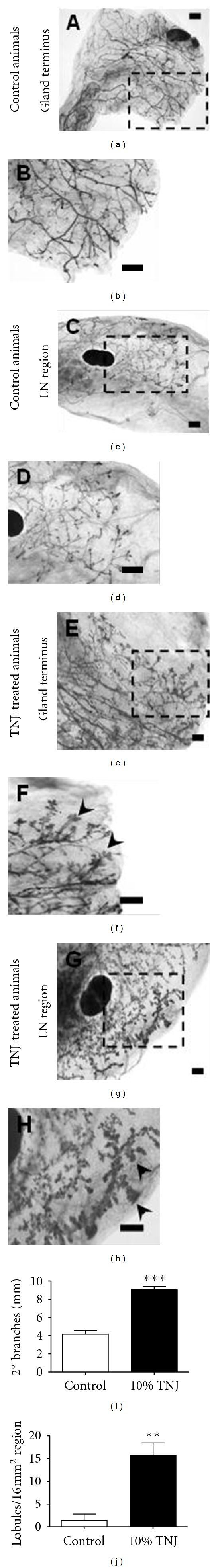
Thirty-day treatment of virgin, tumor-free MMTV-*neu* mice with 10% TNJ results in morphological differences in mammary gland differentiation. Representative photomicrographs shown for 2 animals in the control (panels a–d) and 10% TNJ-treated (panels e–h) groups; animal 1 is shown in panels (a) and (b) for control and (e) and (f) for TNJ groups and animal 2 is shown in panels (c) and (d) for control and (g) and (h) for TNJ groups. For panels (a) and (e) images were captured at the terminus of the gland at magnification 13x; panels (b) and (f) show enlargement of the area outlined by the dashed box at magnification 26x. For panels (c) and (g), images depict the area around the inguinal lymph node to qualitatively assess the degree of ductal branching and lobular differentiation at magnification 13x; panels (d) and (h) are the magnified portion of the gland in the adjacent micrograph outlined by the dashed boxes, magnification 26x. Black arrows identify lobular structures. Inset bars measure 1 mm. The graphs in panels (i) and (j) show quantification of the differentiation evident in the photomicrographs for the mice treated for 30 days with 10% TNJ. (i) The number of secondary ducts was quantified by locating larger, distinct primary ducts and counting the number of secondary branches along a 1-mm distance. Secondary ductule branching in the mammary glands of virgin, tumor-free MMTV-*neu* mice is significantly increased, *P* = 0.0001; control, *n* = 4, and TNJ, *n* = 5. (j) The average number of lobules was determined by quantifying lobuloalveolar structures in four separate 16 mm^2^ areas for each animal. The number of lobules was also significantly increased by TNJ in the same animals analyzed for secondary branching, *P* = 0.0015. Mean ± SEM are shown; TNJ: Tahitian Noni Juice; **indicates significance, *P* < 0.002; ***indicates significance, *P* = 0.0001 by Student's *t-*test.

Ovarian hormones control morphological changes in breast architecture in both humans and rodents [38–40]. In mice, estrogen regulates the elongation of the primary mammary ducts into the mammary fat pad, while progesterone stimulates secondary and tertiary branching and lobuloalveolar development [40]. Qualitative assessment from whole mounts of the inguinal mammary glands of TNJ-treated mice showed a much higher degree of ductal branching and exhibited marked lobuloalveolar development (Figures 7(e)–7(h)) compared to the glands from the control mice (Figures 7(a)–7(d)). To quantify these specific parameters associated with progesterone actions, significantly more secondary ducts along a 1 mm length of the primary duct were detected in the TNJ-treated group compared to the control group (Figure 7(i)). Additionally, the average number of lobuloalveolar structures was significantly higher in the 10% TNJ-treated group (Figure 7(j)). Other analyzed parameters associated with estrogenic activity in the mammary gland, including the number of terminal end buds at the leading edge of the ductal tree, the average size of terminal end bud structures, and the average thickness of primary ducts, were not significantly different between the control and TNJ-treated animals (data not shown). Collectively, the observed responses induced by TNJ treatment (increased ductal arborization and lobule formation) correlate with known progesterone actions even with lower progesterone levels in MMTV-*neu* mice in estrus.

## 4. Discussion

### 4.1. Mammary Tumor Outcomes with TNJ Treatment

TNJ treatment primarily affected mammary tumor growth with no observable effects on tumor latency and incidence in the MMTV-*neu* model of HER2/*neu* breast cancer. These data suggest that, at the dose tested, noni juice may not protect women against the development of HER2^+^ breast cancer. However, it is unknown if higher doses may be able to inhibit MMTV-*neu* mammary and HER2^+^ breast carcinogenesis or if TNJ may have chemopreventative activity against other forms of breast cancer, such as ER^+^ breast tumors. Some previous preclinical cancer studies have injected the noni treatments [4]; however, that delivery method would not compare to oral ingestion by women or account for its potential metabolism by the liver. In the MMTV-*neu* mice, TNJ was supplied orally to correlate to consumption by women. Therefore, if TNJ acts similarly in women and MMTV-*neu* mice, for women taking the recommended dose of TNJ for other disorders or diseases for which noni has been used (such as wound healing, diabetes, high blood pressure, immune stimulation, arthritis, inflammatory disorders, pain, etc.), its lack of effect on mouse mammary tumor development may suggest that this CAM therapy would not increase a woman's risk of developing HER2^+^ breast cancer.

Tumor growth was significantly inhibited in the MMTV-*neu* mice treated with TNJ; the growth reduction of solid tumors was evident in smaller final tumor weights and volumes (Figure 1) and in longer doubling time for large mouse tumors (Figure 2). These findings are in agreement with another study which reported suppressed Ehrlich ascites tumor growth in Balb-c mice treated with noni juice [41]. The most significant growth inhibition in solid mammary tumors from TNJ-treated mice occurred in the size range of 2500–4000 mm^3^ (approximately 1.7 cm to 2.0 cm diameter, Figure 2(d)) and was evident in the weekly tumor growth curves (Figure 2(a)). The growth inhibitory effect of TNJ administration is an important finding given the aggressiveness, hormone-independence, and heterogeneity of tumors in this model. HER2/*neu *overexpression has been implicated in 20–30% of breast cancers and is inversely correlated with patient survival [42, 43]. These tumors are difficult to treat and are most responsive to targeted therapies for the HER2 antigen, such as trastuzumab [44]. The ability of TNJ to inhibit the growth of the mouse tumors, without requiring doses above those recommended for healthy women, suggests that further testing for its potential as a complementary therapy for HER2^+^ breast cancer may be warranted.

### 4.2. Possible Modes of TNJ Mammary Tumor Growth Inhibition

One possible action for the tumor growth inhibition by TNJ may be as an angiogenesis inhibitor. Noni is reported to be a potent inhibitor of angiogenesis by reducing new vessel generation from placental vein explants and inducing degeneration and apoptosis within established capillary networks in human breast tumor explants [11]. The ability of TNJ to inhibit, but to not completely arrest or cause regression of *neu*-induced tumor growth, is consistent with the capabilities of other angiogenesis inhibitors, both in this model and in breast cancer patients [45, 46]. The increase in central necrosis in mammary tumors may allude to antiangiogenic and/or cytotoxic actions of TNJ treatment; however, the lack of a difference in von Willebrand factor immunostaining between control and TNJ-treated tumors does not support this potential mechanism (Table 1). Nonetheless, due to the inherent variability in mammary tumors in this mouse model [47], evidence that other treatments with antiangiogenic actions inhibit tumor growth in *neu* mice [45, 48] and, unlike noni, that antiangiogenic pharmaceuticals are associated with a myriad of side effects [46], further investigation on the possible angiogenic inhibitory actions of noni for suppressing breast tumor growth may be justified.

Tumor growth inhibition by TNJ may also be mediated by immune modulation. Noni is reported to stimulate the immune system [49] and to modify the release of cytokines from immune cells [50, 51]. A case report on two cancer patients taking noni suggested that immune responses may have a role in their long-term survival [52]. Its antitumor and immunomodulatory activities have been linked in preclinical studies using an isolated polysaccharide-rich substance from noni (noni-ppt) in Lewis lung carcinoma model [50, 53] and with a fermented noni exudate (fNE) in mouse sarcoma S180 and Lewis lung carcinoma xenografts [54]. The increased survival of the tumor-bearing mice treated with noni-ppt was blocked with specific inhibitors for macrophages, T-cells, and natural killer cells [50, 53]. For fNE, the pronounced increased survival of fNE-treated C57BL/6J mice injected with S180 cells was reduced in B6 nude mice with a T cell deficiency and was completely blocked in beige mice with a severe natural killer cell deficiency [54]. These studies suggest that these immune cells are essential for inhibiting tumor growth by both noni-derived treatments and, therefore, may also be involved in suppression of MMTV-*neu* mammary tumors. Plus, the MMTV-*neu *mouse model has been documented to be a good model for the immune tolerance observed in HER2/*neu* breast cancer patients [55]. Tolerance allows tumors to grow rapidly and be resistant to death because their cells are not recognized by the immune system. Mechanisms to activate the immune system, such as by inhibiting regulatory T cells, enhanced growth inhibition of implanted *neu*-overexpressing tumors by *neu-*targeted vaccines [56, 57]. Tumor growth in the MMTV-*neu* model with activated *neu* transgene is also suppressed by treatment with cytokines, such as IL-4 and IL-12 [58, 59], which have both been reported to be modified by noni treatments [50, 51]. Therefore, as immunomodulation is effective at reducing tumor growth in *neu* mammary tumor mouse models, this activity of TNJ may be at least partially responsible for inducing similar inhibition in this study.

Other noni activities have been proposed to influence tumor behavior, such as antioxidant and anti-inflammatory actions, which have defined roles in breast cancer [60, 61]. For example, esophageal tumor inhibition by noni resulted in higher serum antioxidant capacity [14]. In a clinical trial, TNJ also reduced superoxide anion radicals and lipid hydroperoxide in smokers [27]. The anti-inflammatory actions of noni, including its ability to directly inhibit COX-2 activity and its corresponding PGE_2_ levels [4, 62], may also be important for the tumor inhibition since COX-2 is critical for *neu*-induced tumorigenesis [63, 64]. Therefore, multiple activities may play a role in the TNJ-induced tumor inhibition in the MMTV-*neu* mice.

Although significant growth inhibitory actions were demonstrated in TNJ-treated mice and histopathology suggests that it inhibits tumor cell invasion into the vascular network in mammary tumors, a corresponding reduction in the incidence of lung micrometastases was not observed. However, these data do not disqualify TNJ as a possible therapy for inhibiting metastatic progression since the HER2/*neu* tumors are aggressive and may require a higher dose than recommended for healthy individuals or tested in this study. In addition, even though it was ineffective alone, TNJ could have a beneficial effect of decreasing metastatic incidence when used in combination with other forms of chemotherapy. Moreover, the lack of effect on metastatic incidence in the MMTV-*neu* mice suggests that TNJ would not increase risk if the primary tumor cells are spreading outside the breast, which would be important for breast cancer patients taking TNJ for other health benefits or continuing problems that preceded cancer. Noni is reported in preclinical and clinical studies to increase energy and quality of life and decrease pain [4, 65, 66]; thus, its use may have potential benefits for cancer patients besides or in addition to its antitumor properties.

### 4.3. Lack of Hepato- and Renal Toxicity by TNJ

TNJ did not elevate serum markers of renal and hepatotoxicity (Figure 5) or change histopathology of either organ in mice treated for 1 year with the mouse equivalent of the recommended dose for humans (<3 oz daily). Previous studies showing lack of toxicity were performed with high doses of noni for shorter time periods, such as 90 mL/kg daily for 3 months in rats [29] and 750 mL/day for 28 days in humans [30, 67]; but, the findings in the MMTV-*neu* mice demonstrate no liver or kidney toxicity with long-term oral administration of a recommended dose. Still, a few clinical case reports have documented that consumption of noni juice can induce reversible hepatotoxicity (except in one case) or hyperkalemia in one patient with chronic renal insufficiency [19–22, 25, 36, 37]. The fact that both liver transaminases were normal in mice in contrast to their elevated levels in the case reports suggests that long-term TNJ consumption did not adversely affect the liver. While the dose of TNJ used in the experimental design is based on the normal recommended dose for consumers, it is important to state that our inbred mouse model exhibits less variability than the human population. Therefore, it is possible that a subset of humans may be susceptible to side effects or adverse events associated with short- or long-term TNJ use. Yet, based on its prevalent use in humans with relatively few reports of toxicity [5], the absence of adverse effects in other clinical and preclinical studies [4, 26, 29, 30, 67], and the lack of toxicity with long-term TNJ administration in MMTV-*neu *mice, noni consumption appears to be safe for the majority of the adult human population.

### 4.4. TNJ Effects on Mammary Gland Differentiation, Hormone Levels, and Reproductive Tract

Enhanced mammary gland differentiation has been inversely correlated with malignant potential of breast epithelial cells. That is, it is proposed that if epithelial cells in undifferentiated lobules never differentiate (e.g., nulliparous women), then they will remain targets of neoplastic transformation [68–70]. Conversely, if differentiation is induced by pregnancy, these epithelial cells become refractory to transformation and have a protective genomic signature against breast cancer development [68–70]. However, in the MMTV-*neu* mice, the increased secondary branching and lobuloalveolar development with TNJ did not result in any protective or adverse effects on mammary tumor development as latency, incidence, and multiplicity were unaffected. But, perhaps the enhanced differentiation in TNJ-treated mouse mammary glands affects the tumors that subsequently develop in these glands to result in slower tumor growth.

The increase in secondary branching and number of lobules with TNJ treatment (Figures 7(i) and 7(j)) are associated with progesterone actions in the mammary gland [40] but are paradoxical to the reduced progesterone levels detected during estrus (Figure 6(e)). Since the hormone levels were not measured during the secretory phase, it is unknown if a corresponding reduction in progesterone also occurs during metestrus and diestrus. If so, possibly the amplitude change between the peak (secretory phase) and nadir (proliferative phase) may be more important than specific progesterone levels and could stimulate enhanced mammary branching and lobule formation. Alternatively, it is possible that the longer time the TNJ-treated mice spent in the secretory phase (Figure 6(a)) may account for the enhanced differentiation that is stimulated in this phase of the estrous cycle.

Noni is reported to have weak estrogenic activity *in vivo *[71], and the flavone glycosides in noni fruit have been classified as phytoestrogens [72]. Thus, the estrogenic components of TNJ and/or the trend to higher 17*β*-estradiol levels in the mice (Figure 6(b)) could potentially upregulate progesterone receptor levels in the uterus and mammary glands of TNJ-treated mice to enhance progesterone sensitivity. However, a uterotropic study in immature rats did not find evidence of estrogenic uterine stimulation by noni extract [73]. The unmodified uterine wet weights in the MMTV-*neu* mice with short- or long-term TNJ treatment coincide with their findings (Figures 6(d) and 6(e)). The study in immature rats reported evidence of antiestrogenicity for noni extract coadministered with 17*α*-ethynylestradiol [73], but this activity was not observed in intact MMTV-*neu* mice as uterine weights were not reduced with TNJ treatment in the presence of endogenous estrogens. Therefore, at the dose tested, estrogenic and/or antiestrogenic properties of TNJ are unlikely to be involved in the uterine and mammary tissue responses in the mice.

Estrous cycling over a 30-day period was significantly modified by TNJ treatment resulting in more days in the secretory phase with a corresponding reduction in the proliferative phase (Figure 6(a)). It is unknown whether this effect is related to the lower progesterone levels. Nonetheless, included in the beneficial claims for noni use is its ability to influence menstrual cycle regulation and cramps, though these benefits lack scientific verification [74]. The altered estrous cycling in MMTV-*neu* mice by TNJ provides the initial scientific support for these claims and suggests that noni may have benefits on women's reproductive health.

### 4.5. TNJ versus Other Noni Products

The results of this study indicate the potential benefits of TNJ on cancer and reproductive outcomes. As there are many different products derived from noni fruit and other parts of the plant, it is unknown if other products would elicit the same effects. One class of active phytochemicals in noni is the iridoids, which has many beneficial activities, including anticancer, antioxidant, anti-inflammatory, and immunomodulating properties [75]. As the iridoid content in noni fruit harvested in different tropical regions and in parts of the noni plant (such as seed, root, flower, and leaf) vary considerably [76], other noni products could result in differing levels of response on the outcomes investigated in the MMTV-*neu* mice. However, as many components of noni juice have been identified which may have beneficial effects [4, 26], the use of noni fruit puree allows the influence of additive and possible synergistic actions of these components versus reductionism to isolated components which are unlikely to have the full benefits of the fruit.

TNJ also contains a small percentage of grape and blueberry juice to enhance palatability, but these juices also contain chemicals, which have anticancer properties [77, 78], have been reported to influence cancer development and/or tumor growth [14, 79–82], and could add or synergize with the noni juice. Thus, it is possible that, even in their low amounts in TNJ, these other juices could contribute to the tumor growth inhibition in the MMTV-*neu* mice. However, in a clinical trial using grape/blueberry juices as a placebo, only TNJ with the noni puree resulted in the beneficial reduction of lipid peroxidation and reactive oxygen species in heavy smokers [27]. Their findings suggest that the small percentage of other juices would not be responsible for the reduced tumor growth in the mice; however, as different outcomes were investigated, these juices may still have a partial role in inhibiting mammary tumor growth.

### 4.6. Summary

TNJ significantly suppressed tumor growth in a model that mimics HER2-overexpressing breast cancer. The fact that these effects occurred using a dose in mice that equates to the recommended human dose is remarkable considering the aggressive and unresponsive nature of HER2/*neu*-positive, hormone-independent breast cancer. Although further studies will be required to determine if TNJ has analogous actions in women as those discovered in this mouse model, tumorigenesis in MMTV-*neu* mice has many similarities to human HER2^+^ breast cancer [16–18]. With the possibility of similar actions in both species, for TNJ use in women with undetected breast cancer, these findings suggest that its growth inhibitory effects may prolong the time until the cancer is diagnosed, as the tumor size at which significant growth suppression was observed (1.5–2 cm in diameter) is within the range of early tumor detection in women (stage 1 breast cancer). For women with HER2^+^ breast cancer, TNJ may suppress tumor growth to allow additional time for surgical or other therapeutic interventions and may enhance the chemotherapy response, as has been reported for noni in rodent models for other types of cancer [41, 53]. Thus, timing, either before or after cancer detection, may ultimately result in improved patient prognosis. Although tested in a hormone-independent model, some of the significant findings reported here for TNJ administration may be applicable to women with hormone-responsive breast cancer and warrant further investigation in appropriate preclinical models. Thus, given the growth inhibitory effects of TNJ on HER2/*neu* tumors and the lack of adverse effects in the MMTV-*neu* model, TNJ may be able to enhance the manageability of HER2^+^ breast cancer to prolong patient survival when administered as a complementary therapy. As more women are seeking natural therapies for general well-being and for treating cancer to reduce the undesired side effects associated with many pharmaceutical options, further clinical and preclinical studies are needed to evaluate the potential of noni for breast cancer treatment and quality of life issues.

## Figures and Tables

**Table 1:** Mammary tumors with vascular invasion and central necrosis detected by histopathology and with blood vessels quantified by von Willebrand factor immunostaining.

	Vascular invasion^a^	Necrotic tumors^a^	Immunostained vessels/20x field^b^
Control	34.5% (10/29)	27.6% (8/29)	2.4 ± 0.2 (*n* = 6)
10% TNJ	13.3% (4/30)	53.3% (16/30)	2.8 ± 0.8 (*n* = 6)

^a^Fisher's exact test, *P* > 0.05.

^b^Mann Whitney test, *P* > 0.05.

**Table 2:** Pulmonary metastases within blood vessels and with extravasion into lung tissue detected by histopathology in tumor-bearing mice.

	Metastatic incidence^a^	Average emboli per mouse^b^	Average invasive lesions per mouse^b^	Average metastases per mouse^b^
Control	70.5% (31/44)	4.4 ± 1.0	4.4 ± 2.5	8.8 ± 1.6
10% TNJ	74.4% (32/43)	4.0 ± 1.1	5.7 ± 1.2	9.7 ± 3.1

^a^Fisher's exact test, *P* > 0.05 for mice with mammary tumors.

^b^Mann-Whitney test, *P* > 0.05 for mice with metastases.
